# Multimodality liver directed treatment for colorectal liver metastasis: Array of complementary options can improve outcomes - A single centre experience from India

**DOI:** 10.3389/fonc.2023.1073311

**Published:** 2023-03-22

**Authors:** Shraddha Patkar, Amit Chopde, Nitin Shetty, Suyash Kulkarni, Kunal Bharat Gala, Daksh Chandra, Anant Ramaswamy, Vikas Ostwal, Mahesh Goel

**Affiliations:** ^1^ GI and HPB Services, Department of Surgical Oncology, Tata Memorial Hospital, Homi Bhabha National Institute, Mumbai, India; ^2^ Department of Interventional Radiology, Tata Memorial Hospital, Homi Bhabha National Institute, Mumbai, India; ^3^ Department of Medical Oncology, Tata Memorial Hospital, Homi Bhabha National Institute, Mumbai, India

**Keywords:** colorectal cancer, colorectal liver metastasis, liver directed surgery, liver resection, multimodality management

## Abstract

**AIM:**

Complimentary use of Liver directed therapies (LDTs) with systemic chemotherapy has improved oncologic outcomes in colorectal liver metastasis (CRLM). We analysed institutional results of multimodality management.

**Methods:**

Retrospective analysis of prospectively maintained database of CRLM patients managed with LDT including surgical resection, Ablation, Transarterial chemoembolization (TACE) or Transarterial radioembolization (TARE) between November 2011 to March 2020. Management plan was decided in multidisciplinary meeting. Resectable tumours underwent surgical resection or ablation or both in some cases. Borderline resectable or unresectable disease was treated with down staging chemotherapy or TACE/TARE followed by resection or ablation. All patients received adjuvant chemotherapy. Factors influencing survival were analysed.

**Results:**

Out of total 375 patients, surgery alone was done in 191 (50.93%) patients while surgery with other LDT in 26 patients (6.93%). Ablation alone was done in 100 (26.66%) whereas TACE/TARE were done as standalone treatment in 21 (5.6%) and 7 (1.86%) patients respectively. TACE + ablation was done in 28 (7.46%) and TARE + ablation was done in 2(0.53%) patients.5-year Overall Survival(OS) was 49.8% while Event free survival(EFS) was 21.4%. The median OS and EFS for surgical group was significantly better than non-surgical group (78 V/s 39 months; p<0.05 and 20 V/s 15 months p <0.005). The resectable (78 months) group had better median OS as compared to borderline resectable and Unresectable group (39 months and 29 months). Male gender, resectable disease and surgical intervention were associated with improved OS.

**Conclusion:**

Although surgery remains the mainstay of treatment, complementary use of non-surgical LDT with systemic therapy offers possibility of good outcomes in advanced liver limited disease. Our experience highlights the impact of multidisciplinary care in optimizing CRLM treatment.

## 1 Introduction

Colorectal cancer (CRC) is the third most common cause of cancer-related deaths in the world with 50%–60% of patients with CRC developing liver metastasis at some point in the disease course (1, 2). Approximately 15%–20% of CRC patients have synchronous colorectal liver metastasis (CRLM) at the time of diagnosis of primary, whereas 30%–40% of patients will develop liver metastasis on follow-up (3). The management of CRLM is challenging, with only 10%–25% of patients having resectable CRC at the time of diagnosis (4, 5). Surgical resection has been considered to be the only curative option for resectable CRLM without extrahepatic disease with a 5-year survival of 40%–50% (6, 7).

Increased use and availability of various treatment modalities in multidisciplinary settings have led to aggressive management of potentially resectable as well as unresectable CRLM with better oncologic outcomes (4, 5, 8, 9). Systemic chemotherapy along with targeted agents has gained an important place in the armamentarium for the management of CRLM (8). Combined use of these liver-directed therapies (LDTs) along with systemic therapy with or without surgery could lead to better overall survival in advanced liver metastatic disease (4, 9). India has a low incidence of CRC compared to the West, but with the increasing availability of diagnostic modalities, an increasing number of cases are being detected (10). The available literature on CRLM from India and South Asia is extremely scarce, and available studies do not focus on the management of CRLM (11, 12). This is one of the earliest experiences from South Asia highlighting the multidisciplinary management of CRLM and the oncologic outcomes.

## 2 Methods

### 2.1 Patient cohort

This study is a retrospective analysis of a prospectively maintained database of all patients undergoing multimodality treatment for CRLM in the form of surgery or other non-surgical LDTs at an eminent tertiary referral centre in India. The period of the study included all patients with CRLM treated from November 2011 to December 2020. Only the patients with a follow-up period of 6 months after the intervention for CRLM were included in the study. The study included all patients with CRLM managed with liver-directed therapies, i.e., surgical resection, radiofrequency ablation (RFA), transarterial chemoembolization (TACE), or transarterial radioembolization (TARE) with or without systemic chemotherapy. Patients treated with palliative intent with only systemic therapy at the index diagnosis as well as patients with extrahepatic metastasis were excluded from the study.

### 2.2 Multidisciplinary treatment

All potential patients of CRLMs were discussed in a multidisciplinary “Liver Clinic” meeting comprising surgical, medical, and radiation oncologists along with interventional radiologists. Demographic parameters and clinico-radiologic data were recorded. Characteristics of the primary tumour, viz., location, stage, treatment modalities, tumour differentiation, and serum carcinoembryonic antigen (CEA) levels, were also noted.

Contrast-enhanced computed tomography (CECT) scan of the thorax, abdomen, and pelvis or whole-body positron emission tomography and computed tomography (PET-CT) was performed to stage and characterize the liver metastases such as size, distribution, i.e., unilobar versus bilobar, and the number of lesions. Depending upon the extent of the disease, tumour location, and liver function, patients were categorized as resectable, borderline resectable, or unresectable (**Table 1**).

**Table 1:** Criteria for resectability of liver disease.

Category	Criteria
Resectable	* All tumour lesions could be removed with adequate margins and FLR > 30% in chemotherapy-naïve patients and FLR > 40% in post-chemotherapy patients * No major intrahepatic vascular involvement precluding safe resection
Borderline resectable	* Size of hepatic lesions >5 cm, >4 metastatic lesions * Proximity to all hepatic veins or both branches of portal vein not amenable for R0 resection upfront * Limited bilobar liver metastasis
Unresectable	* Extensive bilobar liver metastases not amenable for resection * Lesion involving either hepatic hilum or cloaca precluding resection

Surgery was performed with curative intent to obtain a margin negative (R0) resection for patients with resectable disease. For superficial lesions, a non-anatomical resection was performed. For lesions situated deep in the liver parenchyma, a formal liver resection such as segmentectomy or hepatectomy was performed as per the International Hepatopancreatobiliary Association Brisbane 2000 Classification (13).

Synchronous liver metastases were managed with simultaneous resection or a staged approach as per discretion of the surgical team depending on the extent of the disease, the site of primary, and the nature of the liver resection that would be required. The metachronous liver lesions were confirmed as colorectal metastasis by immunohistochemistry.

Some patients, though deemed resectable, underwent ablation, as the tumour location would have entailed the loss of a large amount of normal liver parenchyma if resected. Some were treated with a combined approach of surgery with intraoperative or perioperative ablations.

Patients with borderline resectable and unresectable diseases were treated with downsizing conversion chemotherapy (in combination with other LDTs in few cases) followed by curative intent surgery if adequate downsizing was achieved by chemotherapy to facilitate R0 resection. Those who were still unresectable after chemotherapy received non-surgical LDTs with systemic chemotherapy.

Ablation by RFA or microwaves was performed in few resectable tumours as described above and for unresectable diseases or if patients were either unfit or refused surgery. TACE was used in patients with unresectable liver disease as a means of disease control and also as a downsizing tool in few patients. TARE using yttrium-90 (a pure beta emitter)-impregnated microspheres to the hepatic tumours *via* the tumour arterial feeders were used mainly for those patients who had progressive but liver-limited unresectable disease post-chemotherapy.

### 2.3 Response and follow-up

For patients undergoing ablation, response assessment was performed using a regional PET scan 4–6 weeks and 3 months from the day of ablation using the Response Evaluation Criteria in Solid Tumors (RECIST) criteria (14). Patients treated with TACE and TARE were followed up with PET-CT (with triphasic CECT) after 4 weeks of the procedure. Treatment response was assessed using Positron Emission Tomography Response Criteria in Solid Tumours (PERCIST) criteria and RECIST criteria (14, 15).

All patients were offered post-intervention adjuvant chemotherapy and followed up every 3 months with serum CEA and routine blood investigations and an ultrasound examination of the abdomen with a chest X-ray. A CECT scan of the thorax, abdomen, and pelvis was performed every 6 months for the first 2 years and annually thereafter or when there was suspicion of recurrence. The last follow-up was performed telephonically.

The data of the present study were collected in the course of common clinical practice, and accordingly, signed informed consent was obtained from each patient. Data collection was in accordance with the Declaration of Helsinki. (16) As per institutional protocol, a formal board review was not taken, as it is a retrospective analysis.

### 2.4 Statistical analysis

SPSS version 25 was used for statistical analysis. Student’s t-test was used for quantitative data. The chi-square test was used for categorical variables. Overall survival (OS) and event-free survival (EFS) were calculated using the Kaplan–Meier curves. OS was calculated from the diagnosis date till death due to any cause or last follow-up, and EFS was calculated from the date of intervention for CRLM to either recurrence or progression of the disease. Univariate and multivariate analyses for prognostic variables were performed using the Cox regression analysis.

## 3 Results

### 3.1 Demographics and population details

**Figure 1:**
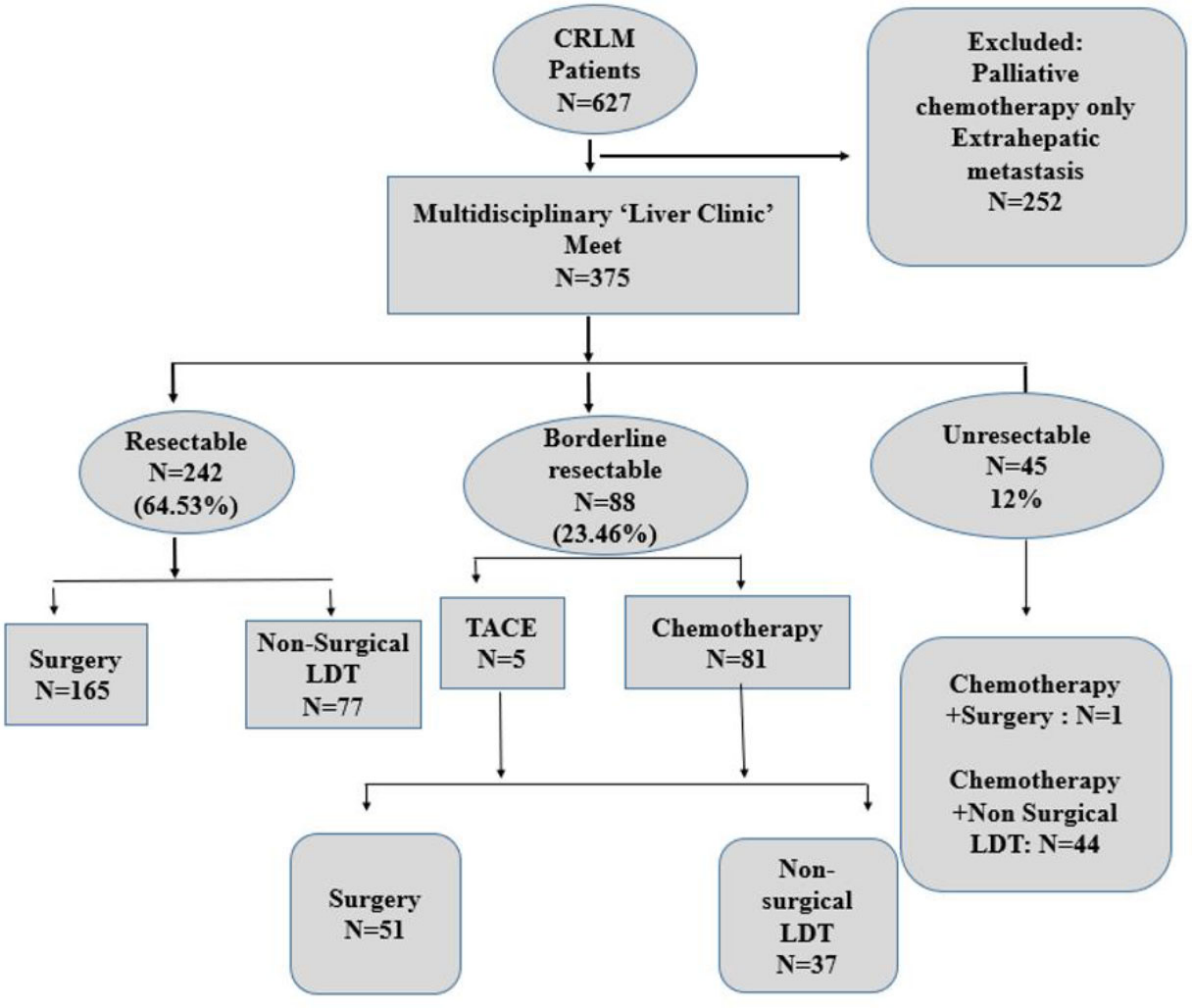
CONSORT diagram depicting the subgroups as per resectability criteria and treatment received.

A total of 627 cases of CRLM were discussed in the multidisciplinary meeting. Out of these, 252 cases were excluded, as they had extrahepatic metastasis or were treated with palliative chemotherapy without any LDT. Finally, approximately 375 eligible patients undergoing multimodality treatment for CRLM including surgical or non-surgical LDTs were included in the study (**Figure 1**). The total cohort consisted of 245 (65.3%) male patients and 130 (34.7%) female patients with a male-to-female ratio of 1.8:1. The median age of the population was 55 (range, 22–82) years.

The primary cancer was addressed with surgical resection in all but four (1.06%) patients who either had progressive metastatic disease even after undergoing liver-directed treatment along with chemotherapy (n = 3) or refused surgery for primary disease (n = 1). The rectum was the most prevalent primary site, with 221 (58.9%) patients. More than four metastatic lesions were seen in 64 (17.1%) patients. **Table 2** describes the characteristics of the primary tumour and liver metastatic disease.

**Table 2:** List of primers used in this study.

Characteristic	Total cohortN (Percentage)	Resectable^*^	Borderline resectable ^*^	Unresectable^*^	P value
Total Number of patients	375	242	88	45	–
Median Age	55 (range: 22-82).	53 (22-78)	57 (26-82)	53(24-81)	0.49
Male : Female	1.8:1	1.8:1	2.3:1	2.2:1	–
Primary Site Right colon and Transverse colon Decending colon Sigmoid colon Rectum	98 (26.13%) 7 (1.86%) 49(13.06%) 221(58.5%)	–	_	_	–
Treatment groups Surgery Non Surgery	217 (57.86%) 158 (42.13%)	165(68.18%) 77 (31.81%)	51 (57.95%) 37 (42.04%)	1 (2.2%) 44 (97.77%)	–
T Stage T1 T2 T3 T4 Tx	42 (11.20%) 53 (14.13%) 221(58.93%) 55 (14.66%) 4 (1.06%)	22 (9.09%) 32(13.32%) 150(61.98%) 36 (14.87%) 2 (0.8%)	11 (12.5%) 12 (13.63%) 51 (57.95%) 14 (15.90%) 0	9 (2%) 9 (2%) 20 (44.44%) 5 (11.11%) 2 (4.44%)	0.086
Nodal stage N0 N1 N2 Nx	109 (29.03%) 231 (61.6%) 31 (8.26%) 4 (1.06%)	69 (28.51%) 147(60.74%) 24 (9.91%) 2 (0.8%)	24 (27.27%) 60 (68.18%) 4 (4.5%) 0	16 (35.55%) 24 (53.33%) 3 (6.66 %) 2 (4.44%)	0.116
kRAS Mutation Wild Type Not Tested	97 (25.86 %) 200(53.30%) 78 (20.8%)	60 (24.79%) 119(49.17%) 63 (26.03%)	25 (28.40%) 50 (56.81%) 13 (14.77%)	12 (26.66%) 31 (68.88%) 2 (4.44%)	–
Multiple liver metastasis (more than 1)	205	84 (34.71%)	77 (87.5%)	44 (97.7%)	0.01
Number of liver lesions ≥ 4	64	2 (0.8%)	35 (39.77%)	27(60%)	<0.01
Mean Size (Cumulative) of lesions	3.58cm ±2.19	3.31 cm ±2.23	4.01 ±2.55	5.6cm ±1.8	0.01
Size of lesion >5 cm	70	5 (2.06%)	26 (29.54%)	39 (86.66%)	<0.01
Distribution Unilobar Bilobar	258 117	227 (93.80%) 15 (6.19%)	31 (35.22%) 57 (64.77%)	0 45 (100%)	<0.01
Neoadjuvant Chemotherapy	237	104 (42.97%)	88 (100%)	45 (100%)	<0.0001
Simultaneous Resections	60 /224 (27.67 %)	60(23.96%)	0	0	
Synchronous Metachronus	247 128	143 (59.09%) 99 (40.90%)	68 (77.27%) 20 (22.72%)	36 (80%) 9 (20%)	0.001
Median CEA	56 (IQR 2-112 )	11.5 IQR (4-42.8)	14 (IQR :6.8-69.74)	77(IQR 11-56)	0.8

CEA, carcinoembryonic antigen.

Synchronous liver metastasis was seen in 247 (65.9%) patients, while 128 (34.1%) developed metachronous liver metastasis. The median time interval between primary surgery and identification of metachronous lesion was 15 months (range, 8 to 123 months). Amongst patients presenting with the synchronous disease, the most common primary site was the rectum at 122/247 (49.34%), followed by the ascending and transverse colon (87; 35.22%) and the left colon (38; 15.38%). Of the patients, 64 (17.1%) developed extrahepatic disease during the follow-up period with the lung (28 patients; 43.75%) as the most common site.

A total of 237 (58.7%) patients received some form of neoadjuvant chemotherapy before any surgical or non-surgical LDT intervention. Most of them were fluoropyrimidine-oxaliplatin-based regimens. Four cycles of chemotherapy were administered prior to surgery in most cases. All patients received adjuvant chemotherapy post-intervention. In addition, based on K-RAS status, targeted cetuximab was given in the later part of the study.

### 3.2 LDTs

Surgical resection was performed in 217 (57.9%) patients as a definitive modality for the CRLM, and 158 (42.1%) patients underwent non-surgical LDTs. Out of 217 patients undergoing surgical modality, 191 (88%) patients underwent only surgery, whereas 21 (9.7%) patients were treated by a combination of surgical resection and post-operative ablation by either RFA or microwave energy. Five (2.3%) patients underwent surgery after being downsized by TACE. Of the total, 92 (42.3%) patients underwent parenchyma-preserving non-anatomical liver resections, with 125 (57.6%) patients requiring a formal hepatic resection.

In the surgical cohort, median blood loss was 750 ml (range, 30–13,000 ml). The median hospital stay was 7 days (range, 3–65 days). The major (Clavien–Dindo Grade III and above) post-operative morbidity was 9.8%, and mortality was 1.8%. Post-operative liver insufficiency and bile leaks were the commonest complications occurring in 6.4% (n = 14) and 5.5% (n = 12) patients, respectively. A total of 60 patients had simultaneous resection of primary tumour along with liver metastasis and had similar post-operative major morbidity rates as compared to staged resection. All patients received chemotherapy after surgery.

**Figure 2:**
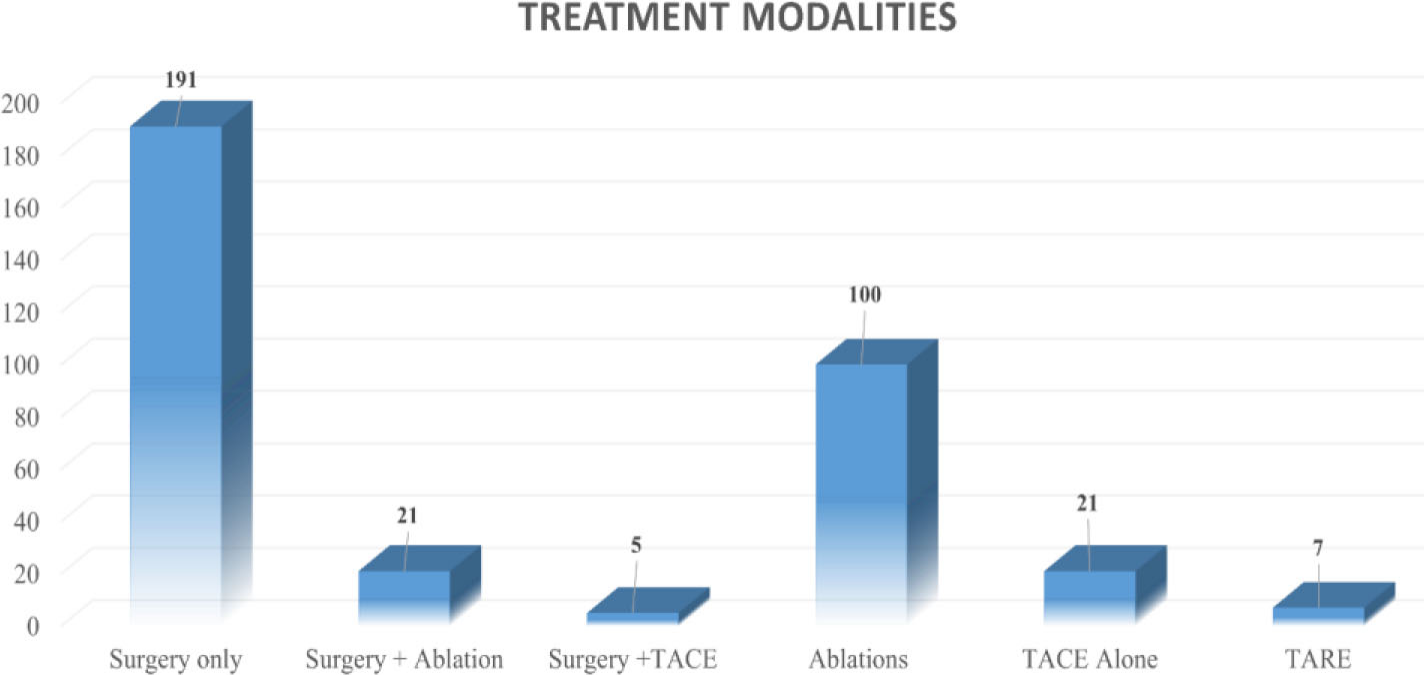
Various treatment modalities.

Amongst patients receiving non-surgical LDT, 100 (63.29%) patients received ablation alone. Of the patients, 49 received TACE either as a standalone treatment option [n = 21; (13.92%)] or in combination with ablation [n = 28 (17.77%)]. Seven (4.4%) patients were managed by TARE as a standalone treatment option, while two were managed with a combination of TARE with ablation. There was no major complication or mortality in the non-surgical group with a median hospital stay of 2.5, 3, and 2.8 days for those undergoing ablation, TACE, and TARE, respectively (**Figure 2**).

### 3.3 Follow-up and survival

At a median follow-up period of 39 (range, 6–163) months, the median OS for the entire study population was 52 months (range, 6–163) months. At the last follow-up, 106 (28.3%) patients were alive and disease free, whereas 70 (18.7%) patients were alive with disease. Of the patients, 158 (42.1%) died due to the disease, and 30 were lost to follow-up (8%).

**Figure 3:**
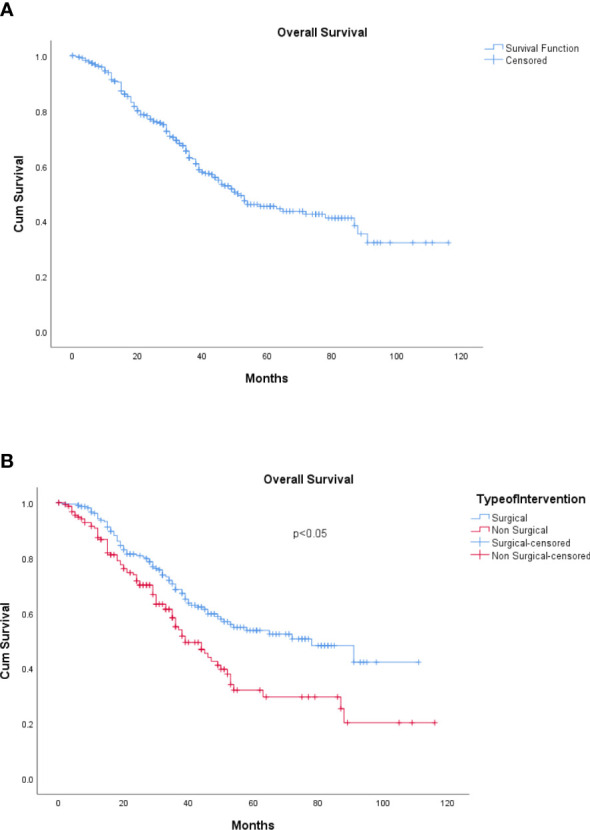
**(A)** Overall survival. **(B)** Overall survival: surgical and non-surgical LDT. LDT, liver-directed therapy.

**Figure 4:**
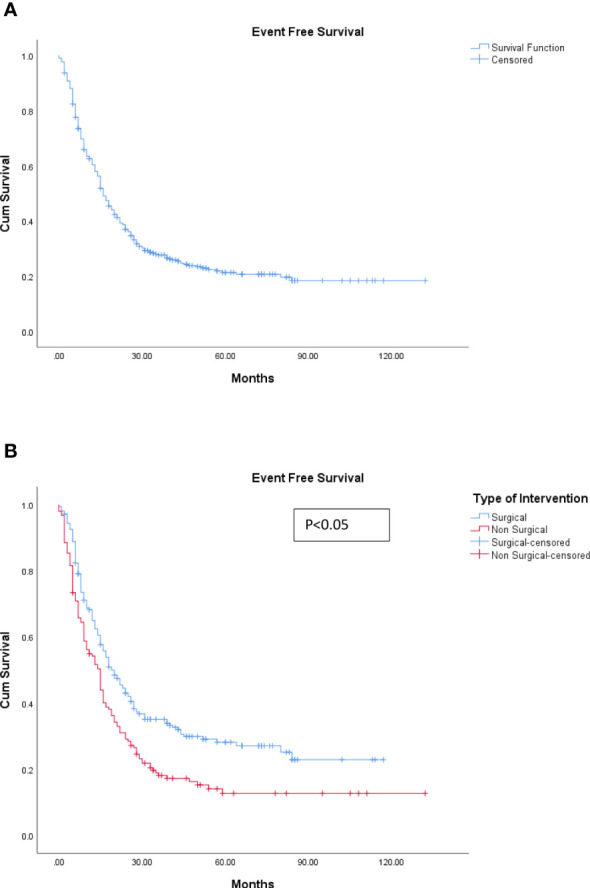
**(A)** Event-free survival. **(B)** Event-free survival: surgical and non-surgical LDT. LDT, liver-directed therapy.

The median EFS was 16 months (range, 0–138 months). The 5-year overall survival for the cohort was 49.8%, whereas the 5-year EFS was 21.4% (refer to **Figures 3A**, **4A**).

The median OS for the patients undergoing surgical resection was 78 months (range, 6–163 months) compared to 39 months (range, 6–142) for those patients who underwent non-surgical LDTs, which was statistically significant (p < 0.05) (refer to **Figure 3B**). Similarly, the median EFS was significantly better in the surgical group (20 months) as compared with the non-surgical LDT group (15 months) (p < 0.005) (refer to **Figure 4B**).

Recurrence was seen in 133 patients out of the 217 undergoing surgical resection (61.2%), while 132 patients had disease progression (83.5%) after non-surgical LDT.

**Figure 5:**
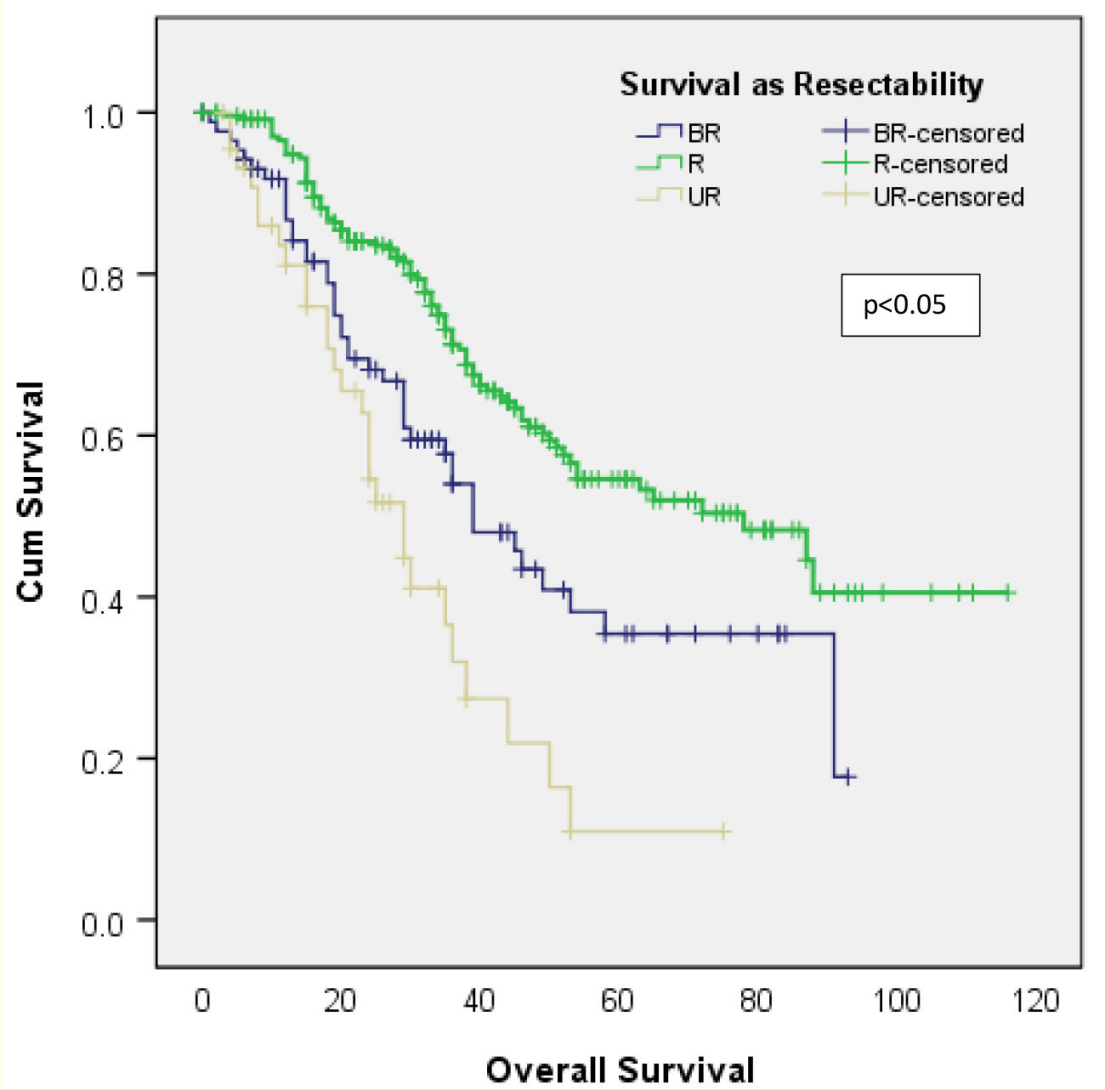
Survival as per resectability. R, resectable; BR, borderline resectable; UR, unresectable.

Better OS was observed in patients considered to have a resectable disease (median OS, 78 months) in the original multidisciplinary meeting as compared to patients with borderline resectable (median OS, 39 months) and unresectable disease (median OS, 29 months). The unresectable group had the worse survival (refer to **Figure 5**).

On univariate analysis, the OS was significantly worse in women as compared to men (0.002). Also, metastatic lesions more than 4 (p = 0.001) bilobar distribution of the metastatic lesions (p = 0.0001), borderline resectable (p = 0.039) as well as an unresectable disease at (p = 0.0001), elevated CEA levels more than 5 ng/ml (p = 0.016), and non-surgical LDT (p = 0001) were associated with poor OS (refer **Table 3**) Age, site of primary, size of tumour >5 cm, KRAS mutational status, synchronous or metachronous liver metastasis, neo-adjuvant or pre-procedural conversion chemotherapy, and T and N stage of disease did not affect OS.

**Table 3:** Factors affecting OS: univariate and multivariate analyses.

Variable	Univariate analysis p-Value	Multivariate analysis p-Value	HR (95% CI)
**Age**	0.615	–	
**Gender**	0.002	0.0001	1.79 (1.30–2.47)
**Site of primary**	0.522	–	
**Bilobar distribution of disease**	0.0001	–	
**Number of lesions >4**	0.001	–	
**Resectability** **Resectable** **Borderline resectable** **Unresectable**	Ref 0.035 0.0001	Ref 0.002 0.0001	1.83 (1.26–2.64) 2.91 (1.80–4.69)
**CEA > 5 ng/ml**	0.016	–	
**Surgical resection**	0.001	0.042	1.43 (1.01–2.02)
**Size > 5 cm**	0.162	–	
**KRAS mutation**	p = 0.40	–	
**T stage** **T1** **T2** **T3** **T4** **Tx**	Ref 0.245 0.943 0.472 0.488	–	
**N stage** **N0** **N1** **N2** **Nx**	Ref 0.390 0.223 0.951	–	
**Neoadjuvant chemotherapy**	0.961	–	
**Synchronous metastasis**	0.101		

OS, overall survival; CEA, carcinoembryonic antigen.

A stepwise multivariate analysis, however, showed that only female gender (HR 1.79, 95% CI 1.30–2.47, p = 0.002), borderline resectable disease (HR 1.83 95% CI 1.26–2.64, p = 0.0001), unresectable disease (HR 2.91 95% CI 1.80 –4.69, p = 0.0001), and non-surgical LDTs as the modality of treatment (HR 1.43, 95% CI 1.01–2.02, p = 0.042) were independently associated with poor OS.

## 4 Discussion

Management of CRLM remains challenging despite the availability of multiple modalities for treatment. With the availability of effective chemotherapy and access to a variety of ablative and non-surgical modalities of LDTs, an increasing number of patients with CRLM are being managed through a combination of the different treatment modalities (17, 18). Though the multimodality approach has become an integral part of treating CRLM, literature exploring multimodality treatment outcomes is limited especially from the South Asian region (11, 12, 19–22). This is the largest series on multimodality liver-directed therapy in CRLM from the Indian sub-continent.

In the present study, the entire cohort has a median OS of 52 months and 5-year overall survival of 49.8%. Similar results for multimodal treatment were reported by other studies (9, 23). We have also found that acceptable disease control in terms of event-free survival could be achieved by complementary use of various surgical as well as non-surgical LDTs (**Table 4**). Extirpation/local control of disease with or without chemotherapy has been shown to have better survival than palliative chemotherapy alone, and our results convey similar findings (5, 9, 23).

**Table 4:** Multimodality treatment of CRLM.

Study	Modalities	Study details Sub-group (n)	Median OS for multimodality arm	Median OS for chemotherapy-only arm
Joharatnam-Hogan N et al. (2020)^23^	Surgery and/or ablation/radiotherapy/TACE with chemotherapy	Multimodal treatment n = 74 Chemotherapy only n = 51	33.6 months	14 months
Ruers T et al. (2017)^9^	Surgery + RFA or RFA alone with chemotherapy	Surgery + RFA n = 60 Chemotherapy only n = 59	45.6 months (5-year OS, 43.1%)	40.5 months
Current study	Surgery + other LDTs or non-surgical LDTs	Surgery n = 217 Non-surgical LDT n = 158	52 months (5-year OS, 49.8%)	NA

CRLM, synchronous colorectal liver metastasis; OS, overall survival; TACE, transarterial chemoembolization; RFA, radiofrequency ablation; LDTs, liver-directed therapies.

Advances in surgical technique and intra-operative management have rendered major hepatectomies safe and expanded the scope of extended resections for CRLMs with the only caveat being adequate functional remnant liver volume (24, 25). Major post-operative morbidity in our series was 9.8%, with 1.8% (n = 4) mortality in the surgery group, which is in keeping with the published literature (24, 25). The 5-year OS for surgically treated patients was 54.5%, which is comparable to that of published literature (5, 26, 27).

The current study has 65% of cases presenting as synchronous colorectal metastasis. This reflects the referral patterns in a developing country with few centres of excellence for the management of metastatic colorectal cancer and may not be representative of the population as a whole. Indeed, most patients of CRLM are referred to us on the diagnosis of liver metastasis for further management leading to skewing of data. Many patients received resection of primary before referral. For synchronous CRLM, the approach of resection of the primary tumour and liver metastasis whether performed simultaneously or staged has not been shown to affect perioperative outcomes (28, 29). In the present study, no difference in peri-operative outcomes was seen in simultaneously resected patients as compared to staged liver resection.

Though modern chemotherapy and biological agents have improved the median survival of advanced CRLM patients up to 15–30 months, the intention remains palliative with historical 5-year survival rates <10% (5, 30–32). In patients with unresectable hepatic disease, conversion chemotherapy is reported to downsize the metastases and convert them to potentially resectable disease (9, 32). The use of chemotherapy, with or without targeted therapy, has resulted in conversion rates to surgery in patients with unresectable liver metastases, but there are unanswered questions regarding the routine use of neoadjuvant therapy in patients with resectable disease. A recent meta-analysis showed the benefit of neoadjuvant chemotherapy (NACT) with increased disease-free survival, but it did not translate into overall survival benefit (33). Similarly, the European Organisation for Research and Treatment of Cancer (EORTC) trial showed increased progression-free survival at 3 years by 9.2% with NACT compared with surgery alone in patients undergoing liver resection (34).

In the present study, NACT was administered to 58.7% of patients prior to LDT. Neoadjuvant chemotherapy in resectable liver metastasis has been treated previously without NACT over the years, especially in the right colonic primary. The majority (242; 64.5%) of the patients in this cohort had resectable liver disease. Resectable liver metastasis has been treated previously without NACT over the years, especially in the right colonic primary. Our practice has evolved over the years from upfront resections to neoadjuvant chemotherapy. We found that, though the use of chemotherapy helped in downsizing and further resection or nonsurgical LDT being given to the patients, neoadjuvant chemotherapy failed to show benefit in improving OS on univariate and multivariate analyses in this study. This finding is consistent with the long-term results of the EORTC trial and other studies (34, 35). The potential benefit of chemotherapy needs to be weighed against the associated hepatotoxicity in evaluating patients with resectable CRLM.

Non-surgical LDTs have traditionally been used for the management of unresectable CRLM either alone or more often in conjunction with systemic chemotherapy. Most of the evidence available for non-surgical LDTs evaluates a single modality, i.e., RFA/ablations, and many of them are single-arm studies [36.37]. We have used non-surgical liver-directed interventions in patients with unresectable or borderline resectable CRLM or in resectable disease patients deemed unfit for surgery or small resectable CRLM, which would have entailed a formal hepatectomy due to their anatomical location.

The OS and EFS for non-surgical LDTs in the current study were significantly less than those for surgically treated patients due to inherent selection bias. The only phase II randomized trial comparing systemic chemotherapy alone versus combination with RFA has shown a clear benefit with a significantly longer progression-free survival as well as OS in the combination arm (9). We had 100 patients who were managed with only ablations and systemic chemotherapy. The median OS in this group was 53 months (6–142 months), which was comparable to that of the surgical group (p = 0.46). This explains the good OS in the non-surgical LDT group and correlates with previous studies (36, 37).

TARE with yttrium-90 (^90^Y)-loaded microspheres has emerged in the last decade as an effective method of local disease control in patients with chemorefractory disease. A phase III randomized trial has shown prolongation in time to liver progression in patients with unresectable chemorefractory CRLM receiving radioembolization with ^90^Y-resin microspheres (38). We used TARE in seven patients who had progressive but liver-limited unresectable disease post-chemotherapy and in two patients with disease progression after RFA. At 3 months’ follow-up, clinical benefit rate (CBR) was 83.33%, suggesting good local disease control.

Various prognostic factors affecting survival like age, gender, site of primary, and KRAS mutation have been described in the literature (39, 40). We found that factors such as male gender, resectable disease, and surgical intervention were associated with better outcomes. KRAS mutational status had no effect on survival in the present study, contradicting other studies (40). However, not all patients had mutational analysis, which could explain this lack of effect.

The current study is limited in its retrospective nature as well as non-uniform systemic chemotherapy received in neoadjuvant and adjuvant settings, which limits generalization. There is also some selection bias in the treatment offered based on the resectability and performance status of patients. Also, the study does not take into account the site of recurrence of CRC whether local or distant and only pertains to patients having liver metastasis without any extrahepatic disease, which may have bearing on the oncologic outcomes. In the future, a focussed prospective study accommodating multimodality treatment could overcome the shortcomings of this study. However, we believe this effort highlights the importance of the multidisciplinary team (MDT) approach reflecting real-world management of CRLM. We have found that extirpation/local control of disease with or without chemotherapy is better than historically shown survival by palliative chemotherapy alone and that acceptable outcomes can be achieved with multimodality treatment even in advanced liver-limited disease. We have also found that whenever feasible, LDT either alone or in combinations can be effectively used in carefully selected patients to achieve good disease control. However, their impact on improving long-term survival remains to be ascertained.

## 5 Conclusion

Aggressive multimodality treatment of CRLM could lead to good oncologic outcomes. Surgical resection remains the mainstay of treatment with better OS and EFS than other modalities. Complementary use of non-surgical options along with systemic therapy offers the possibility of good survival outcomes in unresectable advanced liver-limited diseases. The role of multidisciplinary meetings exploring all available options for CRLM patients is crucial for overall favourable outcomes.

## Data availability statement

The data that support the findings of this study are available from the corresponding author, [MG], upon reasonable request due to ethical and legal restrains.

## Author contributions

Conceptulization: MG. Data collection: SP, AC, NS, KG, DC, SK. Data Analysis: AC, SP, AR, VO. Drafting of manuscript: All. All authors contributed to the article and approved the submitted version.
